# Current Awareness on Comparative and Functional Genomics

**DOI:** 10.1002/cfg.490

**Published:** 2005


**Comparative and Functional Genomics**
*Comp Funct Genom* 2005; **6**: 334–341
Published online in Wiley InterScience (www.interscience.wiley.com). **DOI:** 10.1002cfg.490

### Current awareness on comparative and functional genomics

**Current Awareness – Use the links online**

### 1 Reviews & symposia

2005. Special issue: DNA microarrays and expression profiling in drug abuse research. *Addict Biol* **10:** (1)
2005. Special issue: Functional genomics of ageing II. *Mech Ageing Dev* **126:** (1)
2005. Special issue: Proteomics databases part III. *J Chromatogr B* **815:** (1-2)
2005. Special issue: The Molecular Biology Database Collection: 2005 update. *Nucleic Acids Res* **33:** (Database issue)
Armstrong NJ, Van de Wiel MA. 2004. Microarray data analysis: From hypotheses to conclusions using gene expression data (Review). *Cell Oncol* **26:** (5-6) 279.
Bentley SD, Parkhill J. 2004. Comparative genomic structure of prokaryotes. *Annu Rev Genet* **38:** 771.
Berriman M. 2004. The genomes of eukaryotic pathogens. *S Afr J Sci* **100:** (9-10) 452.
Bolonna AA, Arranz MJ, Mancama D, Kerwin RW. 2004. Pharmacogenomics - Can genetics help in the care of psychiatric patients? *Int Rev Psychiatry* **16:** (4) 311.
Brandt R, Grutzmann R, Bauer A, Jesnowski R, Ringel J, Lohr M, Pilarsky C, Hoheisel JD. 2004. DNA microarray analysis of pancreatic malignancies (Review). *Pancreatology* **4:** (6) 587.
Callanan M. 2005. Mining the probiotic genome: Advanced strategies, enhanced benefits, perceived obstacles. *Curr Pharm Design* **11:** (1) 25.
Duncan MJ. 2003. Genomics of oral bacteria. *Crit Rev Oral Biol Med* **14:** (3) 175.
Ephritikhine G, Ferro M, Rolland N. 2004. Plant membrane proteomics. *Plant Physiol Biochem* **42:** (12) 943.
Ferrando AA, Look AT. 2004. DNA microarrays in the diagnosis and management of acute lymphoblastic leukemia. *Int J Hematol* **80:** (5) 395.
Grossman AR. 2005. Paths toward algal genomics. *Plant Physiol* **137:** (2) 410.
Gunsalus KC, Piano F. 2005. RNAi as a tool to study cell biology: Building genome-phenome bridge. *Curr Opin Cell Biol* **17:** (1) 3.
Hirschhorn JN, Daly MJ. 2005. Genome-wide association studies for common diseases and complex traits. *Nat Rev Genet* **6:** (2) 95.
Hughes S, Arneson N, Done S, Squire J. 2005. The use of whole genome amplification in the study of human disease (Review). *Prog Biophys Mol Biol* **88:** (1) 173.
Kelley J, Walter L, Trowsdale J. 2005. Comparative genomics of major histocompatibility complexes (Review). *Immunogenetics* **56:** (10) 683.
Khabar KSA. 2005. The AU-rich transcriptome: More than interferons and cytokines, and its role in disease (Review). *J Interferon Cytokine Res* **25:** (1) 1.
Kralj M, Kraljevic S, Sedic M, Kurjak A, Pavelic K. 2005. Global approach to perinatal medicine: Functional genomics and proteomics (Review). *J Perinat Med* **33:** (1) 5.
Lange BM, Ghassemian M. 2005. Comprehensive post-genomic data analysis approaches integrating biochemical pathway maps (Review). *Phytochemistry* **66:** (4) 413.
Lee SJ, Saravanan RS, Damasceno CMB, Yamane H, Kim BD, Rose JKC. 2004. Digging deeper into the plant cell wall proteome. *Plant Physiol Biochem* **42:** (12) 979.
Liu ET. 2004. Expression genomics and cancer drug development. *Drug Dev Res* **62:** (4) 295.
Lundstrom K. 2005. Structural genomics of GPCRs (Review). *Trends Biotechnol* **23:** (2) 103.
Millar AH, Heazlewood JL, Kristensen BK, Braun HP, Moller IM. 2005. The plant mitochondrial proteome (Review). *Trends Plant Sci* **10:** (1) 36.
Mockler TC, Ecker JR. 2005. Applications of DNA tiling arrays for whole-genome analysis (Review). *Genomics* **85:** (1) 1.
Posadas EM, Simpkins F, Liotta LA, MacDonald C, Kohn EC. 2005. Proteomic analysis for the early detection and rational treatment of cancer - Realistic hope? (Review). *Ann Oncol* **16:** (1) 16.
Renesto P, Ogata H, Audic S, Claverie JM, Raoult D. 2005. Some lessons from *Rickettsia* genomics. *FEMS Microbiol Rev* **29:** (1) 99.
Resing KA, Ahn NG. 2005. Proteomics strategies for protein identification (Review). *FEBS Lett* **579:** (4) 885.
Righetti PG, Castagna A, Antonioli P, Boschetti E. 2005. Prefractionation techniques in proteome analysis: The mining tools of the third millennium (Review). *Electrophoresis* **26:** (2) 297.
Scarselli M, Giuliani MM, Adu-Bobie J, Pizza M, Rappuoli R. 2005. The impact of genomics on vaccine design (Review). *Trends Biotechnol* **23:** (2) 84.
Schwacke R, Flugge UI, Kunze R. 2004. Plant membrane proteome databases. *Plant Physiol Biochem* **42:** (12) 1023.
Shih W, Chetty R, Tsao MS. 2005. Expression profiling by microarrays in colorectal cancer (Review). *Oncol Rep* **13:** (3) 517.
Stauber EJ, Hippler M. 2004. *Chlamydomonas reinhardtii* proteomics. *Plant Physiol Biochem* **42:** (12) 989.
Tittiger C. 2004. Functional genomics and insect chemical ecology

In order to keep subscribers up-to-date with the latest developments in their field, this current awareness service is provided by John Wiley & Sons and contains newly-published material on comparative and functional genomics. Each bibliography is divided into 16 sections. 1 Reviews & symposia; 2 General; 3 Large-scale sequencing and mapping; 4 Evolutionary genomics; 5 Comparative genomics; 6 Pathways, gene families and regulons; 7 Pharmacogenomics; 8 EST, cDNA and other clone resources; 9 Functional genomics; 10 Transcriptomics; 11 Proteomics; 12 Protein structural genomics; 13 Metabolomics; 14 Genomic approaches to development; 15 Technological advances; 16 Bioinformatics. Within each section, articles are listed in alphabetical order with respect to author. If, in the preceding period, no publications are located relevant to any one of these headings, that section will be omitted.

Copyright © 2005 John Wiley & Sons, Ltd.

(Review). *J Chem Ecol* **30:** (12) 2335.
Van Wijk KJ. 2004. Plastid proteomics. *Plant Physiol Biochem* **42:** (12) 963.
Vanhecke D, Janitz M. 2005. Functional genomics using high-throughput RNA interference. *Drug Discov Today* **10:** (3) 205.
Wang WYS, Barratt BJ, Clayton DG, Todd JA. 2005. Genome-wide association studies: Theoretical and practical concerns. *Nat Rev Genet* **6:** (2) 109.
Wetmore BA, Merrick BA. 2004. Toxicoproteomics: Proteomics applied to toxicology and pathology. *Toxicol Pathol* **32:** (6) 619.

### 3 Large-scale sequencing and mapping

Brayton KA, Kappmeyer LS, Herndon DR, Dark MJ, Tibbals DL, Palmer GH, McGuire TC, Knowles DP. 2005. Complete genome sequencing of *Anaplasma marginale* reveals that the surface is skewed to two superfamilies of outer membrane proteins. *Proc Natl Acad Sci U S A* **102:** (3) 844.
Karaoglu H, Lee CMY, Meyer W. 2005. Survey of simple sequence repeats in completed fungal genomes. *Mol Biol Evol* **22:** (3) 639.
Larsson P, Oyston PCF, Chain P, Chu MC, Duffield M, Fuxelius HH, Garcia E, Halltorp G, Johansson D, Isherwood KE *et al*. 2005. The complete genome sequence of *Francisella tularensis*, the causative agent of tularemia. *Nat Genet* **37:** (2) 153.
Loftus B, Anderson I, Davies R, Alsmark UCM, Samuelson J, Amedeo P, Roncaglia P, Berriman M, Hirt RP, Mann BJ *et al*. 2005. The genome of the protist parasite *Entamoeba histolytica*. *Nature* **433:** (7028) 865.
Parma P, Erra-Pujada M, Feligini M, Greppi G, Enne G. 2004. Water buffalo (*Bubalus bubalis*): Complete nucleotide mitochondrial genome sequence. *DNA Seq* **15:** (5-6) 369.
Prust C, Hoffmeister M, Liesegang H, Wiezer A, Fricke WF, Ehrenreich A, Gottschalk G, Deppenmeier U. 2005. Complete genome sequence of the acetic acid bacterium *Gluconobacter oxydans*. *Nat Biotechnol* **23:** (2) 195.
Seo JS, Chong HY, Park HS, Yoon KO, Jung C, Kim JJ, Hong JH, Kim H, Kim JH, Kil JI *et al*. 2005. The genome sequence of the ethanologenic bacterium *Zymomonas mobilis* ZM4. *Nat Biotechnol* **23:** (1) 63.
Talla E, Anthouard V, Bouchier C, Frangeul L, Dujon B. 2005. The complete mitochondrial genome of the yeast *Kluyveromyces thermotolerans*. *FEBS Lett* **579:** (1) 30.
Van Pittius NCG, Sampson SL, Warren RM, Van Helden PD. 2004. Genome variation in *Mycobacterium tuberculosis*. *S Afr J Sci* **100:** (9-10) 465.

### 4 Evolutionary genomics

Liu MY, Walch H, Wu SP, Grigoriev A. 2005. Significant expansion of exon-bordering protein domains during animal proteome evolution. *Nucleic Acids Res* **33:** (1) 95.
Wicker T, Robertson JS, Schulze SR, Feltus FA, Magrini V, Morrison JA, Mardis ER, Wilson RK, Peterson DG, Paterson AH *et al*. 2005. The repetitive landscape of the chicken genome. *Genome Res* **15:** (1) 126.

### 5 Comparative genomics

Barbier G, Oesterhelt C, Larson MD, Halgren RG, Wilkerson C, Garavito RM, Benning C, Weber APM. 2005. Comparative genomics of two closely related unicellular thermo-acidophilic red algae, *Galdieria sulphuraria* and *Cyanidioschyzon merolae*, reveals the molecular basis of the metabolic flexibility of *Galdieria sulphuraria* and significant differences in carbohydrate metabolism of both algae. *Plant Physiol* **137:** (2) 460.
Bourque G, Zdobnov EM, Bork P, Pevzner PA, Tesler G. 2005. Comparative architectures of mammalian and chicken genomes reveal highly variable rates of genomic rearrangements across different lineages. *Genome Res* **15:** (1) 98.
Cassat J, Dunman P, McAleese F, Murphy E, Projan S, Smeltzer M. 2005. Comparative genomics of *Staphylococcus aureus* musculoskeletal isolates. *J Bacteriol* **187:** (2) 576.
Erill I, Jara M, Salvador N, Escribano M, Campoy S, Barbe J. 2004. Differences in LexA regulon structure among Proteobacteria through *in vivo* assisted comparative genomics. *Nucleic Acids Res* **32:** (22) 6617.
Faraut T. 2005. Comparative mapping of genomes (French). *Biofutur* (251) 37.
Li HY, Pellegrini M, Eisenberg D. 2005. Detection of parallel functional modules by comparative analysis of genome sequences. *Nat Biotechnol* **23:** (2) 253.
Lombardot T, Bauer M, Teeling H, Amann R, Glockner FO. 2005. The transcriptional regulator pool of the marine bacterium *Rhodopirellula baltica* SH $1^T$ as revealed by whole genome comparisons. *FEMS Microbiol Lett* **242:** (1) 137.
Montsant A, Jabbari K, Maheswari U, Bowler C. 2005. Comparative genomics of the pennate diatom *Phaeodactylum tricornutum*. *Plant Physiol* **137:** (2) 500.
Richards S, Liu Y, Bettencourt BR, Hradecky P, Letovsky S, Nielsen R, Thornton K, Hubisz MJ, Chen R, Meisel RP *et al*. 2005. Comparative genome sequencing of *Drosophila pseudoobscura*: Chromosomal, gene, and *cis*-element evolution. *Genome Res* **15:** (1) 1.
Tyler SD, Peters GA, Severini A. 2005. Complete genome sequence of cercopithecine herpesvirus 2 (SA8) and comparison with other simplexviruses. *Virology* **331:** (2) 429.
Venkatesh B, Yap WH. 2005. Comparative genomics using fugu: A tool for the identification of conserved vertebrate *cis*-regulatory elements. *Bioessays* **27:** (1) 100.

### 6 Pathways, gene families and regulons

Biehl A, Richly E, Noutsos C, Salamini F, Leister D. 2005. Analysis of 101 nuclear transcriptomes reveals 23 distinct regulons and their relationship to metabolism, chromosomal gene distribution and co-ordination of nuclear and plastid gene expression. *Gene* **344:** 33.
Okushima Y, Overvoorde PJ, Arima K, Alonson JM, Chen A, Chang C, Ecker JR, Hughes B, Lui A, Nguyen D *et al*. 2005. Functional genomic analysis of the *AUXIN RESPONSE FACTOR* gene family members in *Arabidopsis thaliana*: Unique and overlapping functions of *ARF7* and *AFR19*. *Plant Cell* **17:** (2) 444.

### 7 Pharmacogenomics

Aoki M, Yamamura Y, Noshiro H, Sakai K, Yokota J, Kohno T, Tokino T, Ishida S, Ohyama S, Ninomiya I *et al*. 2005. A full genome scan for gastric cancer. *J Med Genet* **42:** (1) 83.
Bader JE, Deckert CM, Koglin N, Pluder F, Morl K, Koczan D, Thiesen HJ, Beck-Sickinger AG. 2004. From transcription profile to expression: The signaling repertoire of the SK-N-MC neuroepithelioma cell-line. *J Recept Signal Transduct Res* **24:** (4) 257.
Baggerly KA, Morris JS, Edmonson SR, Coombes KR. 2005. Signal in noise: Evaluating reported reproducibility of serum proteomic tests for ovarian cancer. *J Nat Cancer Inst* **97:** (4) 307.
Baker H, Patel V, Molinolo AA, Shillitoe EJ, Ensley JF, Yoo GH, Meneses-Garcia A, Myers JN, El-Naggar AK, Gutkind JS *et al*. 2005. Proteome-wide analysis of head and neck squamous cell carcinomas using laser-capture microdissection and tandem mass spectrometry. *Oral Oncol* **41:** (2) 183.
Ballot E, Marcelo P, Labas V, Doan S, Zamfir O, Chaumeil C, Vinh J, Batellier L. 2004. Proteomic analysis associating two-dimensional electrophoresis and mass spectrometry to identify lacrimal proteins: A case study (French, English Abstract). *J Fr Ophtalmol* **27:** (10) 1141.
Bergman NH, Passalacqua KD, Gaspard R, Shetron-Rama LM, Quackenbush J, Hanna PC. 2005. Murine macrophage transcriptional responses to *Bacillus anthracis* infection and intoxication. *Infect Immun* **73:** (2) 1069.
Bermudez M, Wells D, Malter H, Munne S, Cohen J, Steuerwald NM. 2004. Expression profiles of individual human oocytes using microarray technology. *Reprod Biomed Online* **8:** (3) 325.
Bjorhall K, Miliotis T, Davidsson P. 2005. Comparison of different depletion strategies for improved resolution in proteomic analysis of human serum samples. *Proteomics* **5:** (1) 307.
Blackhall FH, Pintilie M, Wigle DA, Jurisica I, Liu N, Radulovich N, Johnston MR, Keshavjee S, Tsao M. 2004. Stability and heterogeneity

Copyright © 2005 John Wiley & Sons, Ltd. 

of expression profiles in lung cancer specimens harvested following surgical resection. *Neoplasia* **6:** (6) 761.

Buchanan NS, Hamler RL, Leopold PE, Miller FR, Lubman DM. 2005. Mass mapping of cancer cell lysates using two-dimensional liquid separations, electrospray-time of flight-mass spectrometry, and automated data processing. *Electrophoresis* **26:** (1) 248.

Buhimschi IA, Christner R, Buhimschi CS. 2005. Proteomic biomarker analysis of amniotic fluid for identification of intra-amniotic inflammation. *BJOG* **112:** (2) 173.

Buhimschi IA, Buhimschi CS, Christner R, Weiner CP. 2005. Proteomics technology for the accurate diagnosis of inflammation in twin pregnancies. *BJOG* **112:** (2) 250.

Cao YY, Cao YB, Xu Z, Ying K, Li Y, Xie Y, Zhu ZY, Chen WS, Jiang YY. 2005. cDNA microarray analysis of differential gene expression in *Candida albicans* biofilm exposed to farnesol. *Antimicrob Agents Chemother* **49:** (2) 584.

Chen YD, Zheng S, Yu JK, Hu X. 2004. Artificial neural networks analysis of surface-enhanced laser desorption/ionization mass spectra of serum protein pattern distinguishes colorectal cancer from healthy population. *Clin Cancer Res* **10:** (24) 8380.

Cui JW, Wang J, He K, Jin BF, Wang HX, Li W, Kang LH, Hu MR, Li HY, Yu M *et al*. 2005. Two-dimensional electrophoresis protein profiling as an analytical tool for human acute leukemia 2 classification. *Electrophoresis* **26:** (1) 268.

Davies DH, Liang XW, Hernandez JE, Randall A, Hirst S, Mu YX, Romero KM, Nguyen TT, Kalantari-Dehaghi M, Crotty S *et al*. 2005. Profiling the humoral immune response to infection by using proteome microarrays: High-throughput vaccine and diagnostic antigen discovery. *Proc Natl Acad Sci U S A* **102:** (3) 547.

De Mey JGR, Schiffers PM, Hilgers RHP, Sanders MMW. 2005. Toward functional genomics of flow-induced outward remodeling of resistance arteries. *Am J Physiol* **288:** (3) H1022.

Desouza L, Diehl G, Yang ECC, Guo JZ, Rodrigues MJ, Romaschin AD, Colgan TJ, Siu KWM. 2005. Proteomic analysis of the proliferative and secretory phases of the human endometrium: Protein identification and differential protein expression. *Proteomics* **5:** (1) 270.

Eisenhofer G, Huynh TT, Pacak K, Brouwers FM, Walther MM, Linehan WM, Munson PJ, Mannelli M, Goldstein DS, Elkahloun AG. 2004. Distinct gene expression profiles in norepinephrine- and epinephrine-producing hereditary and sporadic pheochromocytomas: Activation of hypoxia-driven angiogenic pathways in von Hippel-Lindau syndrome. *Endocr Relat Cancer* **11:** (4) 897.

Ek S, Ortega E, Borrebaeck CAK. 2005. Transcriptional profiling and assessment of cell lines as *in vitro* models for mantle cell lymphoma. *Leuk Res* **29:** (2) 205.

Freiberg C, Fischer HP, Brunner NA. 2005. Discovering the mechanism of action of novel antibacterial agents through transcriptional profiling of conditional mutants. *Antimicrob Agents Chemother* **49:** (2) 749.

Funding M, Vorum H, Honore B, Nexo E, Ehlers N. 2005. Proteomic analysis of aqueous humour from patients with acute corneal rejection. *Acta Ophthalmol Scand* **83:** (1) 31.

Gagliano N, Moscheni C, Dellavia C, Stabellini G, Ferrario VF, Gioia M. 2005. Immunosuppression and gingival overgrowth: Gene and protein expression profiles of collagen turnover in FK506-treated human gingival fibroblasts. *J Clin Periodontol* **32:** (2) 167.

Geuijen CAW, Bijl N, Smit RCM, Cox F, Throsby M, Visser TJ, Jongeneelen MAC, Bakker ABH, Kruisbeek AM, Goudsmit J *et al*. 2005. A proteomic approach to tumour target identification using phage display, affinity purification and mass spectrometry. *Eur J Cancer* **41:** (1) 178.

Graham MR, Virtaneva K, Porcella SF, Barry WT, Gowen BB, Johnson CR, Wright FA, Musser JM. 2005. Group A *Streptococcus* transcriptome dynamics during growth in human blood reveals bacterial adaptive and survival strategies. *Am J Pathol* **166:** (2) 455.

Greene JG, Dingledine R, Greenamyre JT. 2005. Gene expression profiling of rat midbrain dopamine neurons: Implications for selective vulnerability in parkinsonism. *Neurobiol Dis* **18:** (1) 19.

Grossman HB, Messing E, Soloway M, Tomera K, Katz G, Berger Y, Shen Y. 2005. Detection of bladder cancer using a point-of-care proteomic assay. *JAMA* **293:** (7) 810.

Guajardo JR, Schliefer K, Daines MO, Ruddy RM, Aronow BJ, Wills-Karp M, Hershey GKK. 2005. Altered gene expression profiles in nasal respiratory epithelium reflect stable *versus* acute childhood asthma. *J Allergy Clin Immunol* **115:** (2) 243.

Hemingway J. 2004. The influence of genomics on the control of malaria and other vector-borne diseases. *S Afr J Sci* **100:** (9-10) 475.

Hoehenwarter W, Kumar NM, Wacker M, Zimny-Arndt U, Klose J, Jungblut PR. 2005. Eye lens proteomics: From global approach to detailed information about phakinin and γ E and F crystallin genes. *Proteomics* **5:** (1) 245.

Hu WY, Jones PD, Celius T, Giesy JP. 2005. Identification of genes responsive to PFOS using gene expression profiling. *Environ Toxicol Pharmacol* **19:** (1) 57.

Iwamoto K, Bundo M, Kato T. 2005. Altered expression of mitochondria-related genes in postmortem brains of patients with bipolar disorder or schizophrenia, as revealed by large-scale DNA microarray analysis. *Hum Mol Genet* **14:** (2) 241.

Iwao-Koizumi K, Matoba R, Ueno N, Kim SJ, Ando A, Miyoshi Y, Maeda E, Noguchi S, Kato K. 2005. Prediction of docetaxel response in human breast cancer by gene expression profiling. *J Clin Oncol* **23:** (3) 422.

Jacquemier J, Ginestier C, Rougemont J, Bardou VJ, Charafe-Jauffret E, Geneix J, Adelaide J, Koki A, Houvenaeghel G, Hassoun J *et al*. 2005. Protein expression profiling identifies subclasses of breast cancer and predicts prognosis. *Cancer Res* **65:** (3) 767.

Jamshidi-Parsian A, Dong YB, Zheng XY, Zhou HSS, Zacharias W, McMasters KM. 2005. Gene expression profiling of E2F-1-induced apoptosis. *Gene* **344:** 67.

Jansen MPHM, Foekens JA, Van Staveren IL, Dirkzwager-Kiel MM, Ritstier K, Look MP, Meijer-van Gelder ME, Sieuwerts AM, Portengen H, Dorssers LCJ *et al*. 2005. Molecular classification of tamoxifen-resistant breast carcinomas by gene expression profiling. *J Clin Oncol* **23:** (4) 732.

Jiang YM, Yamamoto M, Kobayashi Y, Yoshihara T, Liang YD, Terao S, Takeuchi H, Ishigaki S, Katsuno M, Adachi H *et al*. 2005. Gene expression profile of spinal motor neurons in sporadic amyotrophic lateral sclerosis. *Ann Neurol* **57:** (2) 236.

Jing Z, Goodlett DR, Peskind ER, Quinn JF, Yong Z, Qin W, Pan C, Yi E, Eng J, Aebersold RH *et al*. 2005. Quantitative proteomic analysis of age-related changes in human cerebrospinal fluid. *Neurobiol Aging* **26:** (2) 207.

Joyner DE, Wade ML, Szabo A, Bastar J, Coffin CM, Albritton KH, Bernard PS, Randall RL. 2005. Discriminate gene lists derived from cDNA microarray profiles of limited samples permit distinguishing mesenchymal neoplasia *ex vivo*. *J Cancer Res Clin Oncol* **131:** (3) 137.

Kamei A, Takamura S, Nagai M, Takeuchi N. 2004. Phosphoproteome analysis of hereditary cataractous rat lens α-crystallin. *Biol Pharm Bull* **27:** (12) 1923.

Kim JM, Sohn HY, Yoon SY, Oh JH, Yang JO, Kim JH, Song KS, Rho SM, Yoo HS, Kim YS *et al*. 2005. Identification of gastric cancer-related genes using a cDNA microarray containing novel expressed sequence tags expressed in gastric cancer cells. *Clin Cancer Res* **11:** (2) 473.

Kim JY, Kwon J, Kim JE, Koh WS, Chung MK, Yoon S, Song CW, Lee M. 2005. Identification of potential biomarkers of genotoxicity and carcinogenicity in L5178Y mouse lymphoma cells by cDNA microarray analysis. *Environ Mol Mutagen* **45:** (1) 80.

Kim TM, Jeong HA, Seo MY, Kim SC, Cho G, Park CH, Kim TS, Park KH, Chung HC, Rha SY. 2005. Determination of genes related to gastrointestinal tract origin cancer cells using a cDNA microarray. *Clin Cancer Res* **11:** (1) 79.

Kobashi-Hashida M, Hguro N, Tsujikawa M, Furukawa T, Furukawa A, Hashida N, Tsujikawa K, Nakai K, Tano Y. 2005. Micro serial analysis of gene expression in normal human choroid and retinal pigment epithelial transcriptomes. *Jpn J Ophthalmol* **49:** (1) 15.

Kohlmann A, Schoch C, Dugas M, Rauhut S, Weninger F, Schnittger S, Kern W, Haferlach T. 2005. Pattern robustness of diagnostic gene expression signatures in leukemia. *Gene Chromosomes Cancer* **42:** (3) 299.

Kristensen VN, Sorlie T, Geisler J, Langerod A, Yoshimura N, Karesen R, Harada N, Lonning PE, Borresen-Dale AL. 2005. Gene expression profiling of breast cancer in relation to estrogen receptor status and estrogen-metabolizing enzymes: Clinical implications. *Clin Cancer Res* **11:** (2 Pt 2 Suppl) 878S.

Lam LT, Davis RE, Pierce J, Hepperle M, Xu YJ, Hottelet M, Nong

Copyright © 2005 John Wiley & Sons, Ltd. 

YH, Wen DY, Adams J, Dang L *et al*. 2005. Small molecule inhibitors of IκB kinase are selectively toxic for subgroups of diffuse large B-cell lymphoma defined by gene expression profiling. *Clin Cancer Res* **11:** (1) 28.
Larrabee PB, Johnson KL, Lai CQ, Ordovas J, Cowan JM, Tantravahi U, Bianchi DW. 2005. Global gene expression analysis of the living human fetus using cell-free messenger RNA in amniotic fluid. *JAMA* **293:** (7) 836.
Li Y, St John MAR, Zhou XF, Kim Y, Sinha U, Jordan RCK, Eisele D, Abemayor E, Elashoff D, Park NH *et al*. 2004. Salivary transcriptome diagnostics for oral cancer detection. *Clin Cancer Res* **10:** (24) 8442.
Liadaki K, Kho AT, Sanoudou D, Schienda J, Flint A, Beggs AH, Kohane IS, Kunkel LM. 2005. Side population cells isolated from different tissues share transcriptome signatures and express tissue-specific markers. *Exp Cell Res* **303:** (2) 360.
Man YG, Zhang Y, Shen T, Zeng X, Tauler J, Mulshine JL, Strauss BL. 2005. cDNA expression profiling reveals elevated gene expression in cell clusters overlying focally disrupted myoepithelial cell layers: Implications for breast tumor invasion. *Breast Cancer Res Treat* **89:** (2) 199.
Mano H. 2004. Stratification of acute myeloid leukemia based on gene expression profiles. *Int J Hematol* **80:** (5) 389.
Matsuzaki Y, Hashimoto S, Fujita T, Suzuki T, Sakurai T, Matsushima K, Kawakami Y. 2005. Systematic identification of human melanoma antigens using serial analysis of gene expression (SAGE). *J Immunother* **28:** (1) 10.
Michiels S, Koscielny S, Hill C. 2005. Prediction of cancer outcome with microarrays: A multiple random validation strategy. *Lancet* **365:** (9458) 488.
Nessling M, Richter K, Schwaenen C, Roerig P, Wrobel G, Wessendorf S, Fritz B, Bentz M, Sinn HP, Radlwimmer B *et al*. 2005. Candidate genes in breast cancer revealed by microarray-based comparative genomic hybridization of archived tissue. *Cancer Res* **65:** (2) 439.
Nishida K, Mine S, Utsunomiya T, Inoue H, Okamoto M, Udagawa H, Hanai T, Mori M. 2005. Global analysis of altered gene expressions during the process of esophageal squamous cell carcinogenesis in the rat: A study combined with a laser microdissection and a cDNA microarray. *Cancer Res* **65:** (2) 401.
Onda M, Emi M, Yoshida A, Miyamoto S, Akaishi J, Asaka S, Mizutani K, Shimizu K, Nagahama M, Ito K *et al*. 2004. Comprehensive gene expression profiling of anaplastic thyroid cancers with cDNA microarray of 25 344 genes. *Endocr Relat Cancer* **11:** (4) 843.
Pawlik TM, Fritsche H, Coombes KR, Xiao LC, Krishnamurthy S, Hunt KK, Pusztai L, Chen JN, Clarke CH, Arun B *et al*. 2005. Significant differences in nipple aspirate fluid protein expression between healthy women and those with breast cancer demonstrated by time-of-flight mass spectrometry. *Breast Cancer Res Treat* **89:** (2) 149.
Pilarsky C, Wenzig M, Specht T, Saeger HD, Grutzmann R. 2004. Identification and validation of commonly overexpressed genes in solid tumors by comparison of microarray data. *Neoplasia* **6:** (6) 744.
Ponnampalam AP, Weston GC, Trajstman AC, Susil B, Rogers PAW. 2004. Molecular classification of human endometrial cycle stages by transcriptional profiling. *Mol Hum Reprod* **10:** (12) 879.
Poon TCW, Hui AY, Chan HLY, Ang IL, Chow SM, Wong N, Sung JJY. 2005. Prediction of liver fibrosis and cirrhosis in chronic hepatitis B infection by serum proteomic fingerprinting: A pilot study. *Clin Chem* **51:** (2) 328.
Poona HF, Farr SA, Thongboonkerd V, Lynn BC, Banks WA, Morley JE, Klein JB, Butterfield DA. 2005. Proteomic analysis of specific brain proteins in aged SAMP8 mice treated with α-lipoic acid: Implications for aging and age-related neurodegenerative disorders. *Neurochem Int* **46:** (2) 159.
Port M, Schmelz HU, Stockinger M, Sparwasser C, Albers P, Pottek T, Abend M. 2005. Gene expression profiling in seminoma and nonseminoma. *J Clin Oncol* **23:** (1) 58.
Prat O, Berenguer F, Malard V, Tavan E, Sage N, Steinmetz G, Quemeneur E. 2005. Transcriptomic and proteomic responses of human renal HEK293 cells to uranium toxicity. *Proteomics* **5:** (1) 297.
Purkayastha A, Ditty SE, Su J, McGraw J, Hadfiels TL, Tibbetts C, Seto D. 2005. Genomic and bioinformatics analysis of HAdV-4, a human adenovirus causing acute respiratory disease: Implications for gene therapy and vaccine vector development. *J Virol* **79:** (4) 2559.
Ransohoff D. 2005. Lessons from controversy: Ovarian cancer screening and serum proteomics. *J Nat Cancer Inst* **97:** (4) 315.
Rehman I, Azzouzi AR, Catto JWF, Allen S, Cross SS, Feeley K, Meuth M, Hamdy FC. 2004. Proteomic analysis of voided urine after prostatic massage from patients with prostate cancer: A pilot study. *Urology* **64:** (6) 1238.
Remmelink M, Mijatovic T, Gustin A, Mathieu A, Rombaut K, Kiss R, Salmon I, Decaestecker C. 2005. Identification by means of cDNA microarray analyses of gene expression modifications in squamous non-small cell lung cancers as compared to normal bronchial epithelial tissue. *Int J Oncol* **26:** (1) 247.
Reuter A, Fortunato D, Garoffo LP, Napolitano L, Scheurer S, Giuffrida MG, Vieths S, Conti AD. 2005. Novel isoforms of Pru av 1 with diverging immunoglobulin E binding properties identified by a synergistic combination of molecular biology and proteomics. *Proteomics* **5:** (1) 282.
Rho S, Kang M, Choi B, Sim D, Lee J, Lee E, Cho C, Oh JW, Park S, Ko S *et al*. 2005. Effects of Yukmijihwang-tang derivatives (YMJd). A memory enhancing herbal extract, on the gene-expression profile in the rat hippocampus. *Biol Pharm Bull* **28:** (1) 87.
Schraders M, Pfundt R, Straatman HMP, Janssen IM, Van Kessel AG, Schoenmakers EFPM, Van Krieken JHJM, Groenen PJTA. 2005. Novel chromosomal imbalances in mantle cell lymphoma detected by genome-wide array-based comparative genomic hybridization. *Blood* **105:** (4) 1686.
Shan L, Yu MS, Snyderwine EG. 2005. Gene expression profiling of chemically induced rat mammary gland cancer. *Carcinogenesis* **26:** (2) 503.
Skvortsov S, Sarg B, Loeffler-Ragg J, Skvortsova I, Lindner H, Ott HW, Lukas P, Illmensee K, Zwierzina H. 2004. Different proteome pattern of epidermal growth factor receptor-positive colorectal cancer cell lines that are responsive and nonresponsive to C225 antibody treatment. *Mol Cancer Ther* **3:** (12) 1551.
Spentzos D, Levine DA, Ramoni MF, Joseph M, Gu XS, Boyd J, Libermann TA, Cannistra SA. 2004. Gene expression signature with independent prognostic significance in epithelial ovarian cancer. *J Clin Oncol* **22:** (23) 4648.
Sugiura H, Ebise H, Tazawa T, Tanaka K, Sugiura Y, Uehara M, Kikuchi K, Kimura T. 2005. Large-scale DNA microarray analysis of atopic skin lesions shows overexpression of an epidermal differentiation gene cluster in the alternative therapy pathway and tack of protective gene expression in the cornified envelope. *Br J Dermatol* **152:** (1) 146.
Sugiyama Y, Farrow B, Murillo C, Li J, Watanabe H, Sugiyama K, Evers BM. 2005. Analysis of differential gene expression patterns in colon cancer and cancer stroma using microdissected tissues. *Gastroenterology* **128:** (2) 480.
Tan YX, Shi LM, Tong WD, Wang C. 2005. Multi-class cancer classification by total principal component regression (TPCR) using microarray gene expression data. *Nucleic Acids Res* **33:** (1) 56.
Thomas SN, Soreghan BA, Nistor M, Sarsoza F, Head E, Yang AJ. 2005. Reduced neuronal expression of synaptic transmission modulator HNK-1/neural cell adhesion molecule as a potential consequence of amyloid β-mediated oxidative stress: A proteomic approach. *J Neurochem* **92:** (4) 705.
VerBerkmoes NC, Hervey WJ, Shah M, Land M, Hauser L, Larimer FW, Van Berkel GJ, Goeringer DE. 2005. Evaluation of "shotgun" proteomics for identification of biological threat agents in complex environmental matrixes: Experimental simulations. *Anal Chem* **77:** (3) 923.
Vlahou A, Fountoulakis A. 2005. Proteomic approaches in the search for disease biomarkers. *J Chromatogr B* **814:** (1) 11.
Vona-Davis L, Vincent T, Zulfiqar S, Jackson B, Riggs D, McFadden DW. 2004. Proteomic analysis of SEG-1 human Barrett's-associated esophageal adenocarcinoma cells treated with keyhole limpet hemocyanin. *J Gastrointest Surg* **8:** (8) 1018.
Wang C, Chelly MR, Chai NN, Tan YX, Hui T, Li HM, Farkas DL, Demetriou AA. 2005. Transcriptomic fingerprinting of bone marrow-derived hepatic $\beta_2m^-$/Thy-$1^+$ stem cells. *Biochem Biophys Res Commun* **327:** (1) 252.
Wang YX, Klijn JGM, Zhang Y, Sieuwerts A, Look MP, Yang F, Talantov D, Timmermans M, Meijer-van Gelder ME, Yu J *et al*. 2005. Gene-expression profiles to predict distant metastasis of lymph-node-negative primary breast cancer. *Lancet* **365:** (9460) 671.
Weissinger EM, Kaiser T, Meert N, De Smet R, Walden M, Mischak H,

Copyright © 2005 John Wiley & Sons, Ltd. 

Vanholder RC. 2004. Proteomics: A novel tool to unravel the patho-physiology of uraemia. *Nephrol Dial Transplant* **19:** (12) 3068.
Wong N, Chan KYY, Macgregor PF, Lai PBS, Squire JA, Beheshti B, Albert M, Leung TWT. 2005. Transcriptional profiling identifies gene expression changes associated with IFN-$\alpha$ tolerance in hepatitis C-related hepatocellular carcinoma cells. *Clin Cancer Res* **11:** (3) 1319.
Wrobel G, Roerig P, Kokocinski F, Neben K, Hahn M, Reifenberger G, Lichter P. 2005. Microarray-based gene expression profiling of benign, atypical and anaplastic meningiomas identifies novel genes associated with meningioma progression. *Int J Cancer* **114:** (2) 249.
Yin CS, Lee HJ, Hong SJ, Chung JH, Koh HG. 2005. Microarray analysis of gene expression in chondrosarcoma cells treated with bee venom. *Toxicon* **45:** (1) 81.
Ylostalo J, Randall AC, Myers TA, Metzger M, Krogstad DJ, Cogswell FB. 2005. Transcriptome profiles of host gene expression in a monkey model of human malaria. *J Infect Dis* **191:** (3) 400.
You JS, Gelfanova V, Knierman MD, Witzmann F, Wang M, Hale JE. 2005. The impact of blood contamination on the proteome of cerebrospinal fluid. *Proteomics* **5:** (1) 290.
Zhang J, Baines CP, Zong CG, Cardwell EM, Wang GW, Vondriska TM, Ping PP. 2005. Functional proteomic analysis of a three-tier PKC$\varepsilon$-Akt-eNOS signaling module in cardiac protection. *Am J Physiol* **288:** (2) H954.
Zhang SR, Fu JL, Zhou ZC. 2005. Changes in the brain mitochondrial proteome of male Sprague-Dawley rats treated with manganese chloride. *Toxicol Appl Pharmacol* **202:** (1) 13.
Zhang Y, Wang TS, Chen WB, Yilmaz O, Park Y, Jung IY, Hackett M, Lamont RJ. 2005. Differential protein expression by *Porphyromonas gingivalis* in response to secreted epithelial cell components. *Proteomics* **5:** (1) 198.
Zhou P, Kalakonda N, Comenzo RL. 2005. Changes in gene expression profiles of multiple myeloma cells induced by arsenic trioxide (ATO): Possible mechanisms to explain ATO resistance *in vivo*. *Br J Haematol* **128:** (5) 636.
Zucchi I, Mento E, Kuznetsov VA, Scotti M, Valsecchi V, Simionati B, Vicinanza E, Valle G, Pilotti S, Reinbold R *et al*. 2004. Gene expression profiles of epithelial cells microscopically isolated from a breast-invasive ductal carcinoma and a nodel metastasis. *Proc Natl Acad Sci U S A* **101:** (52) 18147.

### 8 EST, cDNA and other clone resources

Anderson JV, Delseny M, Fregene MA, Jorge V, Mba C, Lopez C, Restrepo S, Soto M, Piegu B, Verdier V *et al*. 2004. An EST resource for cassava and other species of Euphorbiaceae. *Plant Mol Biol* **56:** (4) 527.
Chen L, Zhao LP, Gao QK. 2005. Generation and analysis of expressed sequence tags from the tender shoots cDNA library of tea plant (*Camellia sinensis*). *Plant Sci* **168:** (2) 359.
Coles ND, Coleman CE, Christensen SA, Jellen EN, Stevens MR, Bonifacio A, Rojas-Beltran JA, Fairbanks DJ, Maughan PJ. 2005. Development and use of an expressed sequenced tags library in quinoa (*Chenopodium quinoa* Willd.) for the discovery of single nucleotide polymorphisms. *Plant Sci* **168:** (2) 439.
Jansen G, Wu CL, Schade B, Thomas DY, Whiteway M. 2005. Drag&Drop cloning in yeast. *Gene* **344:** 43.
Lichtenzveig J, Scheuring C, Dodge J, Abbo S, Zhang HB. 2005. Construction of BAC and BIBAC libraries and their applications for generation of SSR markers for genome analysis of chickpea, *Cicer arietinum* L. *Theor Appl Genet* **110:** (3) 492.
Lopez C, Jorge V, Piegu B, Mba C, Cortes D, Restrepo S, Soto M, Laudie M, Berger C, Cooke R *et al*. 2004. A unigene catalogue of 5700 expressed genes in cassava. *Plant Mol Biol* **56:** (4) 541.
Park S, Oh S, Han KH. 2004. Large-scale computational analysis of poplar ESTs reveals the repertoire and unique features of expressed genes in the poplar genome. *Mol Breeding* **14:** (4) 429.

### 9 Functional genomics

Dent R, Haglund C, Chin B, Kobayashi M, Niyogi K. 2005. Functional genomics of eukaryotic photosynthesis using insertional mutagenesis of *Chlamydomonas reinhardtii*. *Plant Physiol* **137:** (2) 545.
Gonzalez-Ballester D, De Montaigu A, Higuera JJ, Galvan A, Fernandez E. 2005. Functional genomics of the regulation of the nitrate assimilation pathway in *Chlamydomonas*. *Plant Physiol* **137:** (2) 522.
Lesuisse E, Knight SAB, Courel M, Santos R, Camadro JM, Dancis A. 2005. Genome-wide screen for genes with effects on distinct iron uptake activities in *Saccharomyces cerevisiae*. *Genetics* **169:** (1) 107.
Li YW, Kelly WG, Logsdon JM, Schurko AM, Harfe BD, Hill-Harfe KL, Kahn RA. 2004. Functional genomic analysis of the ADP-ribosylation factor family of GTPases: Phylogeny among diverse eukaryotes and function in *C. elegans*. *FASEB J* **18:** (15) 1834.
Luesch H, Wu TYH, Ren PD, Gray NS, Schultz PG, Supek F. 2005. A genome-wide overexpression screen in yeast for small-molecule target identification. *Chem Biol* **12:** (1) 55.
Medigue C. 2005. Annotating bacterial genomes (French). *Biofutur* (251) 23.
Puttini S, Ouvrard-Pascaud A, Palais G, Beggah AT, Gascard P, Cohen-Tannoudji M, Babinet C, Blot-Chabaud M, Jaisser F. 2005. Development of a targeted transgenesis strategy in highly differentiated cells: A powerful tool for functional genomic analysis. *J Biotechnol* **116:** (2) 145.

### I0 Transcriptomics

Allemeersch J, Durinck S, Vanderhaeghen R, Alard P, Maes R, Seeuws K, Bogaert T, Coddens K, Deschouwer K, Van Hummelen P *et al*. 2005. Benchmarking the CATMA microarray. A novel tool for *Arabidopsis* transcriptome analysis. *Plant Physiol* **137:** (2) 588.
Astua-Monge G, Freitas-Astua J, Bacocina G, Roncoletta J, Carvalho SA, Machado MA. 2005. Expression profiling of virulence and pathogenicity genes of *Xanthomonas axonopodis* pv. *citri*. *J Bacteriol* **187:** (3) 1201.
Bisova K, Krylov DM, Umen JG. 2005. Genome-wide annotation and expression profiling of cell cycle regulatory genes in *Chlamydomonas reinhardtii*. *Plant Physiol* **137:** (2) 475.
Colosimo ME, Brown A, Mukhopadhyay S, Gabel C, Lanjuin AE, Samuel ADT, Sengupta P. 2004. Identification of thermosensory and olfactory neuron-specific genes *via* expression profiling of single neuron types. *Curr Biol* **14:** (24) 2245.
Dieck HT, Doring F, Fuchs D, Roth HP, Daniel H. 2005. Transcriptome and proteome analysis identifies the pathways that increase hepatic lipid accumulation in zinc-deficient rats. *J Nutr* **135:** (2) 199.
Ewart KV, Belanger JC, Williams J, Karakach T, Penny S, Tsoi SCM, Richards RC, Douglas SE. 2005. Identification of genes differentially expressed in Atlantic salmon (*Salmo salar*) in response to infection by *Aeromonas salmonicida* using cDNA microarray technology. *Dev Comp Immunol* **29:** (4) 333.
Fang Y, Choi D, Searles RP, Mathers WD. 2005. A time course microarray study of gene expression in the mouse lacrimal gland after acute corneal trauma. *Invest Ophthalmol Vis Sci* **46:** (2) 461.
Goetz RM, Fuglsang A. 2005. Correlation of codon bias measures with mRNA levels: Analysis of transcriptome data from *Escherichia coli*. *Biochem Biophys Res Commun* **327:** (1) 4.
Hubbard SJ, Grafham DV, Beattie KJ, Overton IM, McLaren SR, Croning MDR, Boardman PE, Bonfield JK, Burnside J, Davies RM *et al*. 2005. Transcriptome analysis for the chicken based on 19,626 finished cDNA sequences and 485,337 expressed sequence tags. *Genome Res* **15:** (1) 174.
Ishii A, Oshima T, Sato T, Nakasone K, Mori H, Kato C. 2005. Analysis of hydrostatic pressure effects on transcription in *Escherichia coli* by DNA microarray procedure. *Extremophiles* **9:** (1) 65.
Jiang ZH, Wu XL, Garcia MD, Griffin KB, Michal JJ, Ott TL, Gaskins CT, Wright RW. 2004. Comparative gene-based *in silico* analysis of transcriptomes in different bovine tissues and (or) organs. *Genome* **47:** (6) 1164.
Kang YS, Weber KD, Yu Q, Kiley PJ, Blattner FR. 2005. Genome-wide expression analysis indicates that FNR of *Escherichia coli* K-12 regulates a large number of genes of unknown function. *J Bacteriol* **187:** (3) 1135.
Lee LJ, Barrett JA, Poole RK. 2005. Genome-wide transcriptional response of chemostat-cultured *Escherichia coli* to zinc. *J Bacteriol* **187:** (3) 1124.
Lopez-Hellin J, Gonzalo R, Tejeda M, Carrascal M, Vila MR, Abian J, Garcia-Arumi E. 2005. Transcriptomic and proteomic analysis of liver

Copyright © 2005 John Wiley & Sons, Ltd. 

and muscle alterations caused by surgical stress in rats. *Clin Sci* **108:** (2) 167.
Moyle R, Fairbairn DJ, Ripi J, Crowe M, Botella JR. 2005. Developing pineapple fruit has a small transcriptome dominated by metallothionein. *J Exp Bot* **56:** (409) 101.
Nagata T, Yamada H, Du ZJ, Todoriki S, Kikuchi S. 2005. Microarray analysis of genes that respond to γ-irradiation in *Arabidopsis*. *J Agric Food Chem* **53:** (4) 1022.
Nielsen KL, Gronkjaer K, Welinder KG, Emmersen J. 2005. Global transcript profiling of potato tuber using LongSAGE. *Plant Biotechnol J* **3:** (2) 175.
Osorio CG, Crawford JA, Michalski J, Martinez-Wilson H, Kaper JB, Camilli A. 2005. Second-generation recombination-based *in vivo* expression technology for large-scale screening for *Vibrio cholerae* genes induced during infection of the mouse small intestine. *Infect Immun* **73:** (2) 972.
Paszek P, Lipniacki T, Brasier AR, Tian B, Nowak DE, Kimmel M. 2005. Stochastic effects of multiple regulators on expression profiles in eukaryotes. *J Theor Biol* **233:** (3) 423.
Polen I, Kramer A, Bongaerts J, Wubbolts M, Wendisch VF. 2005. The global gene expression response of *Escherichia coli* to L-phenylalanine. *J Biotechnol* **115:** (3) 221.
Robbens S, Khadaroo B, Camasses A, Derelle E, Ferraz C, Inze D, Van de Peer Y, Moreau H. 2005. Genome-wide analysis of core cell cycle genes in the unicellular green alga *Ostreococcus tauri*. *Mol Biol Evol* **22:** (3) 589.
Scharf M, Wu-Scharf D, Zhou X, Pittendrigh B, Bennett G. 2005. Gene expression profiles among immature and adult reproductive castes of the termite *Reticulitermes flavipes*. *Insect Mol Biol* **14:** (1) 31.
Scherl A, Francois P, Bento M, Deshusses JM, Charbonnier Y, Converset W, Huyghe A, Walter N, Hoogland C, Appel RD *et al.* 2005. Correlation of proteomic and transcriptomic profiles of *Staphylococcus aureus* during the post-exponential phase of growth. *J Microbiol Methods* **60:** (2) 247.
Seltmann M, Horsch M, Drobyshev A, Chen YL, De Angelis MH, Beckers J. 2005. Assessment of a systematic expression profiling approach in ENU-induced mouse mutant lines. *Mamm Genome* **16:** (1) 1.
Semenova E, Djordjevic M, Shraiman B, Severinov K. 2005. The tale of two RNA polymerases: Transcription profiling and gene expression strategy of bacteriophage Xp10. *Mol Microbiol* **55:** (3) 764.
Vandepoele K, Van De Peer Y. 2005. Exploring the plant transcriptome through phylogenetic profiling. *Plant Physiol* **137:** (1) 31.
Winterberg KM, Luecke J, Bruegl AS, Reznikoff WS. 2005. Phenotypic screening of *Escherichia coli* K-12 Tn5 insertion libraries, using whole-genome oligonucleotide microarrays. *Appl Environ Microbiol* **71:** (1) 451.

### II Proteomics

Aro EM, Suorsa M, Rokka A, Allahverdiyeva Y, Paakkarinen V, Saleem A, Battchikova N, Rintamaki E. 2005. Dynamics of photosystem II: A proteomic approach to thylakoid protein complexes. *J Exp Bot* **56:** (411) 347.
Barreiro C, Gonzalez-Lavado E, Brand S, Tauch A, Martin JF. 2005. Heat shock proteome analysis of wild-type *Corynebacterium glutamicum* ATCC 13032 and a spontaneous mutant lacking GroEL1, a dispensable chaperone. *J Bacteriol* **187:** (3) 884.
Boudart G, Jamet E, Rossignol M, Lafitte C, Borderies G, Jauneau A, Esquerre-Tugaye MT, Pont-Lezica R. 2005. Cell wall proteins in apoplastic fluids of *Arabidopsis thaliana* rosettes: Identification by mass spectrometry and bioinformatics. *Proteomics* **5:** (1) 212.
Cardol P, Gonzalez-Halphen D, Reyes-Prieto A, Baurain D, Matagne RF, Remacle C. 2005. The mitochondrial oxidative phosphorylation proteome of *Chlamydomonas reinhardtii* deduced from the genome sequencing project. *Plant Physiol* **137:** (2) 447.
Charmont S, Jamet E, Pont-Lezica R, Canut H. 2005. Proteomic analysis of secreted proteins from *Arabidopsis thaliana* seedlings: Improved recovery following removal of phenolic compounds. *Phytochemistry* **66:** (4) 453.
Cutillas PR, Biber J, Marks J, Jacob R, Stieger B, Cramer R, Waterfield M, Burlingame AL, Unwin RJ. 2005. Proteomic analysis of plasma membrane vesicles isolated from the rat renal cortex. *Proteomics* **5:** (1) 101.
Dziembowski A, Ventura AP, Rutz B, Caspary F, Faux C, Halgand F, Laprevote O, Seraphin B. 2004. Proteomic analysis identifies a new complex required for nuclear pre-mRNA retention and splicing. *EMBO J* **23:** (24) 4847.
Jorge I, Navarro RM, Lenz C, Ariza D, Porras C, Jorrin J. 2005. The holm oak leaf proteome: Analytical and biological variability in the protein expression level assessed by 2-DE and protein identification tandem mass spectrometry *de novo* sequencing and sequence similarity searching. *Proteomics* **5:** (1) 222.
Klein C, Garcia-Rizo C, Bisle B, Scheffer B, Zischka H, Pfeiffer F, Siedler F, Oesterhelt D. 2005. The membrane proteome of *Halobacterium salinarum*. *Proteomics* **5:** (1) 180.
Konishi H, Kitano H, Komatsu S. 2005. Identification of rice root proteins regulated by gibberellin using proteome analysis. *Plant Cell Environ* **28:** (3) 328.
Korbel S, Buchse T, Prietzsch H, Sasse T, Schumann M, Krause E, Brock J, Bittorf T. 2005. Phosphoprotein profiling of erythropoietin receptor-dependent pathways using different proteomic strategies. *Proteomics* **5:** (1) 91.
Laugesen S, Bergoin A, Rossignol M. 2004. Deciphering the plant phosphoproteome: Tools and strategies for a challenging task. *Plant Physiol Biochem* **42:** (12) 929.
Lebeau I, Lammertyn E, De Buck E, Maes L, Geukens N, Van Mellaert L, Arckens L, Anne J, Clerens S. 2005. First proteomic analysis of *Legionella pneumophila* based on its developing genome sequence. *Res Microbiol* **156:** (1) 119.
Lee PS, Lee KH. 2005. Engineering HlyA hypersecretion in *Escherichia coli* based on proteomic and microarray analyses. *Biotechnol Bioeng* **89:** (2) 195.
Man P, Novak P, Cebecauer M, Horvath O, Fiserova A, Havlicek V, Bezouska K. 2005. Mass spectrometric analysis of the glycosphingolipid-enriched microdomains of rat natural killer cells. *Proteomics* **5:** (1) 113.
Matis M, Zakelj-Mavric M, Peter-Katalinic J. 2005. Mass spectrometry and database search in the analysis of proteins from the fungus *Pleurotus ostreatus*. *Proteomics* **5:** (1) 67.
Murata Y, Doi T, Taniguchi H, Fujiyoshi Y. 2005. Proteomic analysis revealed a novel synaptic proline-rich membrane protein (PRR7) associated with PSD-95 and NMDA receptor. *Biochem Biophys Res Commun* **327:** (1) 183.
Nally JE, Whitelegge JP, Aguilera R, Pereira MM, Blanco DR, Lovett MA. 2005. Purification and proteomic analysis of outer membrane vesicles from a clinical isolate of *Leptospira interrogans* serovar Copenhageni. *Proteomics* **5:** (1) 144.
Pedra JHF, Festucci-Buselli RA, Sun WL, Muir WM, Scharf ME, Pittendrigh BR. 2005. Profiling of abundant proteins associated with dichlorodiphenyltrichloroethane resistance in *Drosophila melanogaster*. *Proteomics* **5:** (1) 258.
Rakwal R, Komatsu S. 2004. Abscisic acid promoted changes in the protein profiles of rice seedling by proteome analysis. *Mol Biol Rep* **31:** (4) 217.
Riccardi F, Gazeau P, Jacquemot MP, Vincent D, Zivy M. 2004. Deciphering genetic variations of proteome responses to water deficit in maize leaves. *Plant Physiol Biochem* **42:** (12) 1003.
Schauder S, Penna L, Ritton A, Manin C, Parker F, Renauld-Mongenie G. 2005. Proteomics analysis by two-dimensional differential gel electrophoresis reveals the lack of a broad response of *Neisseria meningitidis* to *in vitro*-produced AI-2. *J Bacteriol* **187:** (1) 392.
Strey CW, Winters MS, Markiewski MM, Lambris JD. 2005. Partial hepatectomy induced liver proteome changes in mice. *Proteomics* **5:** (1) 318.
Subramanian B, Bansal VK, Kav NNV. 2005. Proteome-level investigation of *Brassica carinata*-derived resistance to *Leptosphaeria maculans*. *J Agric Food Chem* **53:** (2) 313.
Tebbe A, Klein C, Bisle B, Siedler F, Scheffer B, Garcia-Rizo C, Wolfertz J, Hickmann V, Pfeiffer F, Oesterhelt D. 2005. Analysis of the cytosolic proteome of *Halobacterium salinarum* and its implication for genome annotation. *Proteomics* **5:** (1) 168.
Untalan PM, Guerrero FD, Haines LR, Pearson TW. 2005. Proteome analysis of abundantly expressed proteins from unfed larvae of the cattle tick, *Boophilus microplus*. *Insect Biochem Mol Biol* **35:** (2) 141.
Watt SA, Wilke A, Patschkowski T, Niehaus K. 2005. Comprehensive analysis of the extracellular proteins from *Xanthomonas campestris* pv. *campestris* B100. *Proteomics* **5:** (1) 153.

Copyright © 2005 John Wiley & Sons, Ltd. 

Whitelegge JP. 2004. Mass spectrometry for high throughput quantitative proteomics in plant research: Lessons from thylakoid membranes. *Plant Physiol Biochem* **42:** (12) 919.

Yan SP, Tang ZC, Su W, Sun WN. 2005. Proteomic analysis of salt stress-responsive proteins in rice root. *Proteomics* **5:** (1) 235.

Zhang CC, Wei JF, Zheng ZG, Ying NJ, Sheng DH, Hua YJ. 2005. Proteomic analysis of *Deinococcus radiodurans* recovering from γ-irradiation. *Proteomics* **5:** (1) 138.

### I3 Metabolomics

Nikolaev EV, Burgard AP, Maranas CD. 2005. Elucidation and structural analysis of conserved pools for genome-scale metabolic reconstructions. *Biophys J* **88:** (1) 37.

Uchiyama T, Abe T, Ikemura T, Watanabe K. 2005. Substrate-induced gene-expression screening of environmental metagenome libraries for isolation of catabolic genes. *Nat Biotechnol* **23:** (1) 88.

### I4 Genomic approaches to development

Arima K, Shiotsugu J, Niu R, Khandpur R, Martinez M, Shin Y, Koide T, Cho KWY, Kitayama A, Ueno N *et al*. 2005. Global analysis of RAR-responsive genes in the *Xenopus* neurula using cDNA microarrays. *Dev Dyn* **232:** (2) 414.

Chiao E, Leonard J, Dickinson K, Baker JC. 2005. High through-put functional screen of mouse gastrula cDNA libraries reveals new components of endoderm and mesoderm specification. *Genome Res* **15:** (1) 44.

Dauphinot L, Lyle R, Rivals I, Dang MT, Moldrich RX, Golfier G, Ettwiller L, Toyama K, Rossier J, Personnaz L *et al*. 2005. The cerebellar transcriptome during postnatal development of the Ts1Cje mouse, a segmental trisomy model for Down syndrome. *Hum Mol Genet* **14:** (3) 373.

Edwards RG. 2004. Transcriptomes and the analysis of mouse embryo, stem cells and tissue stem cells. *Reprod Biomed Online* **8:** (3) 358.

Guedes SD, Vitorino R, Domingues R, Tomer K, Correia AJF, Amado F, Domingues P. 2005. Proteomics of immune-challenged *Drosophila melanogaster* larvae hemolymph. *Biochem Biophys Res Commun* **328:** (1) 106.

Mukhopadhyay P, Greene RM, Zacharias W, Weinrich MC, Singh S, Young WW, Pisano MM. 2004. Developmental gene expression profiling of mammalian, fetal orofacial tissue. *Birth Defects Res Part A Clin Mol Teratol* **70:** (12) 912.

Peiffer DA, Von Bubnoff A, Shin Y, Kitayama A, Mochii M, Ueno N, Cho KWY. 2005. A *Xenopus* DNA microarray approach to identify novel direct BMP target genes involved in early embryonic development. *Dev Dyn* **232:** (2) 445.

Shastry S, Tyagi N, Hayden MR, Tyagi SC. 2004. Proteomic analysis of homocysteine inhibition of microvascular endothelial cell angiogenesis. *Cell Mol Biol (Noisy-le-Grand)* **50:** (8) 931.

Shin Y, Kitayama A, Koide T, Peiffer DA, Mochii M, Liao A, Ueno N, Cho KWY. 2005. Identification of neural genes using *Xenopus* DNA microarrays. *Dev Dyn* **232:** (2) 432.

Uittenbogaard M, Chiaramello A. 2004. Expression profiling upon Nex1/MATH-2-mediated neuritogenesis in PC12 cells and its implication in regeneration. *J Neurochem* **91:** (6) 1332.

Wen CM, Zhang ZH, Ma WP, Xu M, Wen ZL, Peng JR. 2005. Genome-wide identification of female enriched genes in zebrafish. *Dev Dyn* **232:** (1) 171.

Yamashita Y, Shimada M, Harimoto N, Tanaka S, Shirabe K, Ijima H, Nakazawa K, Fukuda J, Funatsu K, Maehara Y. 2004. cDNA microarray analysis in hepatocyte differentiation in Huh 7 cells. *Cell Transplant* **13:** (7-8) 793.

### I5 Technological advances

Ahram M, Adkins JN, Auberry DL, Wunschel DS, Springer DL. 2005. A proteomic approach to characterise protein shedding. *Proteomics* **5:** (1) 123.

Colinge J, Chiappe D, Lagache S, Moniatte M, Bougueleret L. 2005. Differential proteomics *via* probabilistic peptide identification scores. *Anal Chem* **77:** (2) 596.

Drakas R, Prisco M, Baserga R. 2005. A modified tandem affinity purification tag technique for the purification of protein complexes in mammalian cells. *Proteomics* **5:** (1) 132.

Dufresne-Martin G, Lemay JF, Lavigne P, Klarskov K. 2005. Peptide mass fingerprinting by matrix-assisted laser desorption ionization mass spectrometry of proteins detected by immunostaining on nitrocellulose. *Proteomics* **5:** (1) 55.

Essader AS, Cargile BJ, Bundy JL, Stephenson JL. 2005. A comparison of immobilized pH gradient isoelectric focusing and strong-cation-exchange chromatography as a first dimension in shotgun proteomics. *Proteomics* **5:** (1) 24.

Feuerstein I, Morandell S, Stecher G, Huck CW, Stasyk T, Huang HL, Teis D, Huber LA, Bonn GK. 2005. Phosphoproteomic analysis using immobilized metal ion affinity chromatography on the basis of cellulose powder. *Proteomics* **5:** (1) 46.

Guillemin I, Becker M, Ociepka K, Friauf E, Nothwang HG. 2005. A subcellular prefractionation protocol for minute amounts of mammalian cell cultures and tissue. *Proteomics* **5:** (1) 35.

Hu ZY, Troester M, Perou CM. 2005. High reproducibility using sodium hydroxide-stripped long oligonucleotide DNA microarrays. *Biotechniques* **38:** (1) 121.

Issaq HJ, Chan KC, Janini GM, Conrads TP, Veenstra TD. 2005. Multidimensional separation of peptides for effective proteomic analysis. *J Chromatogr B* **817:** (1) 35.

Jin VX, Leu YW, Liyanarachchi S, Sun H, Fan M, Nephew KP, Huang THM, Davuluri RV. 2004. Identifying estrogen receptor α target genes using integrated computational genomics and chromatin immunoprecipitation microarray. *Nucleic Acids Res* **32:** (22) 6627.

Masselon C, Pasa-Tolic L, Tolic N, Anderson GA, Bogdanov B, Vilkov AN, Shen YF, Zhao R, Qian WJ, Lipton MS, Camp DG, Smith RD. 2005. Targeted comparative proteomics by liquid chromatography-tandem Fourier ion cyclotron resonance mass spectrometry. *Anal Chem* **77:** (2) 400.

Mirzaei H, Regnier F. 2005. Structure specific chromatographic selection in targeted proteomics. *J Chromatogr B* **817:** (1) 23.

Nelson PT, Baldwin DA, Scearce LM, Oberholtzer JC, Tobias JW, Mourelatos Z. 2004. Microarray-based, high-throughput gene expression profiling of microRNAs. *Nat Methods* **1:** (2) 155.

Nielsen PS, Ohlsson H, Alsbo C, Andersen MS, Kauppinen S. 2005. Expression profiling by oligonucleotide microarrays spotted on coated polymer slides. *J Biotechnol* **116:** (2) 125.

Sakai J, Kojima S, Yanagi K, Kanaoka M. 2005. $^{18}$O-labeling quantitative proteomics using an ion trap mass spectrometer. *Proteomics* **5:** (1) 16.

Schaupp CJ, Jiang GJ, Myers TG, Wilson MA. 2005. Active mixing during hybridization improves the accuracy and reproducibility of microarray results. *Biotechniques* **38:** (1) 117.

Schmidt A, Kellermann J, Lottspeich F. 2005. A novel strategy for quantitative proteomics using isotope-coded protein labels. *Proteomics* **5:** (1) 4.

Thomson JM, Parker J, Perou CM, Hammond SM. 2004. A custom microarray platform for analysis of microRNA gene expression. *Nat Methods* **1:** (1) 47.

Venable JD, Dong MQ, Wohlschlegel J, Dillin A, Yates JR. 2004. Automated approach for quantitative analysis of complex peptide mixtures from tandem mass spectra. *Nat Methods* **1:** (1) 39.

Vinarov DA, Lytle BL, Peterson FC, Tyler EM, Volkman BF, Markley JL. 2004. Cell-free protein production and labeling protocol for NMR-based structural proteomics. *Nat Methods* **1:** (2) 149.

Wheeler AR, Moon H, Bird CA, Loo RRO, Kim CJ, Loo JA, Garrell RL. 2005. Digital microfluidics with in-line sample purification for proteomics analyses with MALDI-MS. *Anal Chem* **77:** (2) 534.

Zhong HY, Marcus SL, Li L. 2004. Two-dimensional mass spectra generated from the analysis of $^{15}$N-labeled and unlabeled peptides for efficient protein identification and *de novo* peptide sequencing. *J Proteome Res* **3:** (6) 1155.

### I6 Bioinformatics

Andersson A, Bernander R, Nilsson P. 2005. Dual-genome primer design for construction of DNA microarrays. *Bioinformatics* **21:** (3) 325.

Belshaw R, Katzourakis A. 2005. *BlastAlign*: A program that uses *blast*

Copyright © 2005 John Wiley & Sons, Ltd.

to align problematic nucleotide sequences. *Bioinformatics* **21:** (1) 122.
Carey VJ, Gentry J, Whalen E, Gentleman R. 2005. Network structures and algorithms in Bioconductor (Review). *Bioinformatics* **21:** (1) 135.
Chen LN, Zhou TS, Tang Y. 2005. Protein structure alignment by deterministic annealing. *Bioinformatics* **21:** (1) 51.
Chiappetta P, Roubaud MC, Torresani B. 2004. Blind source separation and the analysis of microarray data. *J Comput Biol* **11:** (6) 1090.
Chou KC. 2005. Using amphiphilic pseudo amino acid composition to predict enzyme subfamily classes. *Bioinformatics* **21:** (1) 10.
Costa IG, De Carvalho FDT, De Souto MCP. 2004. Comparative analysis of clustering methods for gene expression time course data. *Genet Mol Biol* **27:** (4) 623.
Curk T, Demsar J, Xu QK, Leban G, Petrovic U, Bratko I, Shaulsky G, Zupan B. 2005. Microarray data mining with visual programming. *Bioinformatics* **21:** (3) 396.
De Roos ADG. 2005. Origins of introns based on the definition of exon modules and their conserved interfaces. *Bioinformatics* **21:** (1) 2.
Dejori M, Stetter M. 2004. Identifying interventional and pathogenic mechanisms by generative inverse modeling of gene expression profiles. *J Comput Biol* **11:** (6) 1135.
Ein-Dor L, Kela I, Getz G, Givol D, Domany E. 2005. Outcome signature genes in breast cancer: Is there a unique set? *Bioinformatics* **21:** (2) 171.
Etherington GJ, Dicks J, Roberts IN. 2005. Recombination Analysis Tool (RAT): A program for the high-throughput detection of recombination. *Bioinformatics* **21:** (3) 278.
Facius A, Englbrecht C, Birzele F, Groscurth A, Schmid B, Wanka S, Mewes W. 2005. PRIME: A graphical interface for integrating genomic/proteomic databases. *Proteomics* **5:** (1) 76.
Firth AE, Brown CM. 2005. Detecting overlapping coding sequences with pairwise alignments. *Bioinformatics* **21:** (3) 282.
Fu WJJ, Dougherty ER, Mallick B, Carroll RJ. 2005. How many samples are needed to build a classifier: A general sequential approach. *Bioinformatics* **21:** (1) 63.
Haft DH, Selengut JD, Brinkac LM, Zafar N, White O. 2005. Genome Properties: A system for the investigation of prokaryotic genetic content for microbiology, genome annotation and comparative genomics. *Bioinformatics* **21:** (3) 293.
Hakamada K, Hanai T, Honda H, Kobayashi T. 2004. Preprocessing method for inferring genetic interaction from gene expression data using Boolean algorithm. *J Biosci Bioeng* **98:** (6) 457.
Homayouni R, Heinrich K, Wei L, Berry MW. 2005. Gene clustering by Latent Semantic Indexing of MEDLINE abstracts. *Bioinformatics* **21:** (1) 104.
Ihmeis JH, Bergmann S. 2004. Challenges and prospects in the analysis of large-scale gene expression data. *Brief Bioinform* **5:** (4) 313.
Kahraman A, Avramov A, Nashev LG, Popov D, Ternes R, Pohlenz HD, Weiss B. 2005. PhenomicDB: A multi-species genotype/phenotype database for comparative phenomics. *Bioinformatics* **21:** (3) 418.
Karp NA, Griffin JL, Lilley KS. 2005. Application of partial least squares discriminant analysis to two-dimensional difference gel studies in expression proteomics. *Proteomics* **5:** (1) 81.
Kellner WA, Sullivan RT, Carlson BH, Thomas JW. 2005. Uprobe: A genome-wide universal probe resource for comparative physical mapping in vertebrates. *Genome Res* **15:** (1) 166.
Kim H, Golub GH, Park H. 2005. Missing value estimation for DNA microarray gene expression data: Local least squares imputation. *Bioinformatics* **21:** (2) 187.
Kuiken C, Yusim K, Boykin L, Richardson R. 2005. The Los Alamos hepatitis C sequence database. *Bioinformatics* **21:** (3) 379.
Leban G, Bratko I, Petrovic U, Curk T, Zupan B. 2005. VizRank: Finding informative data projections in functional genomics by machine learning. *Bioinformatics* **21:** (3) 413.
Leone M, Pagnani A. 2005. Predicting protein functions with message passing algorithms. *Bioinformatics* **21:** (2) 239.
Lu CL, Huang YP. 2005. A memory-efficient algorithm for multiple sequence alignment with constraints. *Bioinformatics* **21:** (1) 20.
Lu Y, Zhu J, Liu PY. 2005. A two-step strategy for detecting differential gene expression in cDNA microarray data. *Curr Genet* **47:** (2) 121.
Marchin M, Kelly PT, Fang JW. 2005. Tracker: Continuous HMMER and BLAST searching. *Bioinformatics* **21:** (3) 388.
Martin DP, Williamson C, Posada D. 2005. RDP2: Recombination detection and analysis from sequence alignments. *Bioinformatics* **21:** (2) 260.
Martin S, Roe D, Faulon JL. 2005. Predicting protein-protein interactions using signature products. *Bioinformatics* **21:** (2) 218.
Mester DI, Ronin YI, Nevo E, Korol AO. 2004. Fast and high precision algorithms for optimization in large-scale genomic problems. *Comput Biol Chem* **28:** (4) 281.
Nakhamchik A, Zhao ZY, Provart NJ, Shiu SH, Keatley SK, Cameron RK, Goring DR. 2004. A comprehensive expression analysis of the *Arabidopsis* proline-rich extensin-like receptor kinase gene family using bioinformatic and experimental approaches. *Plant Cell Physiol* **45:** (12) 1875.
Ng P, Wei CL, Sung WK, Chiu KP, Lipovich L, Ang CC, Gupta S, Shahab A, Ridwan A, Wong CH *et al*. 2005. Gene identification signature (GIS) analysis for transcriptome characterization and genome annotation. *Nat Methods* **2:** (2) 105.
Ou HY, Smith R, Lucchini S, Hinton J, Chaudhuri RR, Pallen M, Barer MR, Rajakumar K. 2005. ArrayOme: A program for estimating the sizes of microarray-visualized bacterial genomes - Online art. No. e3. *Nucleic Acids Res* **33:** (1) e3.
Reverter A, McWilliam SM, Barris W, Dalrymple BP. 2005. A rapid method for computationally inferring transcriptome coverage and microarray sensitivity. *Bioinformatics* **21:** (1) 80.
Schneider M, Tognolli M, Bairoch A. 2004. The Swiss-Prot protein knowledgebase and ExPASy: Providing the plant community with high quality proteomic data and tools. *Plant Physiol Biochem* **42:** (12) 1013.
Shin SW, Kim SM. 2005. A new algorithm for detecting low-complexity regions in protein sequences. *Bioinformatics* **21:** (2) 160.
Sumazin P, Chen GX, Hata N, Smith AD, Zhang T, Zhang MQ. 2005. DWE: Discriminating Word Enumerator. *Bioinformatics* **21:** (1) 31.
Takahashi H, Kobayashi T, Honda H. 2005. Construction of robust prognostic predictors by using projective adaptive resonance theory as a gene filtering method. *Bioinformatics* **21:** (2) 179.
Tang XY, Shen LS, Dickerson JA. 2005. BarleyExpress: A web-based submission tool for enriched microarray database annotations. *Bioinformatics* **21:** (3) 399.
Tebbutt SJ, Opushnyev IV, Tripp BW, Kassamali AM, Alexander WL, Andersen MI. 2005. SNP Chart: An integrated platform for visualization and interpretation of microarray genotyping data. *Bioinformatics* **21:** (1) 124.
Vucetic S, Obradovic Z, Vacic V, Radivojac P, Peng K, Iakoucheva LM, Cortese MS, Lawson JD, Brown CJ, Sikes JG *et al*. 2005. DisProt: A database of protein disorder. *Bioinformatics* **21:** (1) 137.
Weckx S, De Rijk P, Van Broeckhoven C, Del-Favero J. 2005. SNPbox: A modular software package for large-scale primer design. *Bioinformatics* **21:** (3) 385.
Wesche PL, Gaffney DJ, Keightley PD. 2004. DNA sequence error rates in Genbank records estimated using the mouse genome as a reference. *DNA Seq* **15:** (5-6) 362.
Yang ZR. 2004. Biological applications of support vector machines. *Brief Bioinform* **5:** (4) 328.
Ye L, Huang XQ. 2005. MAP2: Multiple alignment of syntenic genomic sequences. *Nucleic Acids Res* **33:** (1) 162.
Yiu SM, Wong PWH, Lam TW, Mui YC, Kung HF, Lin M, Cheung YT. 2005. Filtering of ineffective siRNAs and improved siRNA design tool. *Bioinformatics* **21:** (2) 144.
Young A, Whitehouse N, Cho J, Shaw C. 2005. OntologyTraverser: An R package for GO analysis. *Bioinformatics* **21:** (2) 275.
Zhang K, Qin ZH, Chen T, Li JS, Waterman MS, Sun FZ. 2005. HapBlock: Haplotype block partitioning and tag SNP selection software using a set of dynamic programming algorithms. *Bioinformatics* **21:** (1) 131.
Zhou LQ, Yu ZG, Deng JQ, Anh V, Long SC. 2005. A fractal method to distinguish coding and non-coding sequences in a complete genome based on a number sequence representation. *J Theor Biol* **232:** (4) 559.
Zhou XHJ, Kao MCJ, Huang HY, Wong A, Nunez-Iglesias J, Primig M, Aparicio OM, Finch CE, Morgan TE, Wong WH. 2005. Functional annotation and network reconstruction through cross-platform integration of microarray data. *Nat Biotechnol* **23:** (2) 238.
Zou M, Conzen SD. 2005. A new dynamic Bayesian network (DBM) approach for identifying gene regulatory networks from time course microarray data. *Bioinformatics* **21:** (1) 71.

Copyright © 2005 John Wiley & Sons, Ltd. 
